# Drugged Driving among Sexual Minority Older Adults in the United States, 2015-2019

**DOI:** 10.1093/geroni/igab046.2778

**Published:** 2021-12-17

**Authors:** Jie Yang, Andrew Yockey

**Affiliations:** 1 East Carolina University, Greenville, North Carolina, United States; 2 University of North Texas Health Science Center, Denton, Texas, United States

## Abstract

Introduction: Drugged driving, the act of operating vehicles under the influence of one or more illicit substances is responsible for numerous emergency department visits, deaths, and increased medical costs. Despite higher instances of drug use, older sexual minority (LGB) adults are often neglected in prevention efforts. This study assessed inequities between sexual minority older adults and their heterosexual counterparts in drugged driving across three difference substances (alcohol, marijuana, other drugs). Methods: Pooled data from individuals 50 years or older (n = 43,238; 1,115 sexual minority adults) in the 2015–2019 National Survey on Drug Use and Health were analyzed. Past-year driving under the influence of alcohol, marijuana, and illicit drugs were outcome variables, and survey-weighted frequencies with 95% confidence limits and crude ORs with 95% confidence intervals (CI) were estimated. Results: In the past year, 4.82% of older adults drove under the influence of alcohol, 1.33% drove under the influence of marijuana, and 1.48% drove under the influence of illicit drugs. Sexual minority adults consistently showed higher odds of drugged driving than their heterosexual counterparts, with individuals who identify as bisexual being most at risk for driving under the influence of illicit drugs (aOR:4.49, 95%CI: 2.84, 7.08) and marijuana (aOR:3.95, 95%CI: 2.39, 6.51). Discussion: There are differences drugged driving by sexual orientation across the three substances we assessed. These rates of substance use among older sexual minority adults warrant ongoing concern, and it is critical to consider differences across the life course in designing and evaluating interventions to address inequities.

